# Sero-epidemiological study of arbovirus infection following the 2015–2016 Zika virus outbreak in Cabo Verde

**DOI:** 10.1038/s41598-022-16115-4

**Published:** 2022-07-09

**Authors:** Daniel Ward, Ana Rita Gomes, Kevin K. A. Tetteh, Nuno Sepúlveda, Lara Ferrero Gomez, Susana Campino, Taane G. Clark

**Affiliations:** 1 Department of Infection Biology, London School of Hygiene and Tropical Medicine, Keppel Street, London, WC1E 7HT UK; 2 Université de Montpellier, Montpellier, France; 3 Warsaw University of Technology, Warsaw, Poland; 4 Universidade de Lisboa, Lisbon, Portugal; 5 Universidade Jean Piaget (UniPiaget), Praia, Cabo Verde

**Keywords:** Risk factors, Viral infection, Epidemiology

## Abstract

In November 2015, cases of Zika virus infection were recorded in Cabo Verde (Africa), originating from Brazil. The outbreak subsided after seven months with 7580 suspected cases. We performed a serological survey (n = 431) in Praia, the capital city, 3 months after transmission ceased. Serum samples were screened for arbovirus antibodies using ELISA techniques and revealed seroconverted individuals with Zika (10.9%), dengue (1–4) (12.5%), yellow fever (0.2%) and chikungunya (2.6%) infections. Zika seropositivity was predominantly observed amongst females (70%). Using a logistic model, risk factors for increased odds of Zika seropositivity included age, self-reported Zika infection, and dengue seropositivity. Serological data from Zika and dengue virus assays were strongly correlated (Spearman’s r_s_ = 0.80), which reduced when using a double antigen binding ELISA (Spearman’s r_s_ = 0.54). Overall, our work improves an understanding of how Zika and other arboviruses have spread throughout the Cabo Verde population. It also demonstrates the utility of serological assay formats for outbreak investigations.

## Introduction

In February 2016, a public health emergency of international concern was declared due to a prolonged Zika virus epidemic (ZIKV) spreading rapidly across the Americas. There were infections implicated in causing microcephaly or congenital Zika syndrome manifestations in 2–50% of children exposed prenatally^1,2^. The spread of ZIKV also coincided with an increase in the incidence of Guillain–Barré syndrome^3,4^. Before the arrival and spread of ZIKV across the Americas^5–7^, outbreaks were reported in the Pacific islands of Yap (73% of population exposed)^8^, then French Polynesia (2013–2014; 30k cases; 11.5% of population), followed by further cases on New Caledonia, the Cook Islands and Easter Island^9,10^. Phylogenetic analysis revealed that early Brazilian strains are ancestral to a French Polynesian strain (KJ776791), indicating that the ZIKV circulating in the Americas was most likely exported from the Pacific island region^11^. Before the end of year 2015, eleven countries in the Americas had reported PCR positive ZIKV cases. As the cases of ZIKV climbed in South America, a lesser-reported intercontinental transmission event was suspected in November 2015, on Cabo Verde, an archipelago located off the coast of Senegal, Africa, with strong economic, travel and tourism links to Brazil. To date, at least 87 countries worldwide have reported ZIKV transmission^12^, with > 580k suspected cases of infection^13^.

ZIKV is a positive-sense single stranded RNA virus, with a 10.8 kbp non-segmented genome. It belongs to the genus *Flavivirus,* which includes common human arboviruses such as dengue virus (DENV), West Nile virus (WNV) and yellow fever virus (YFV), which alongside the *Alphavirus* chikungunya (CHIKV), are transmitted by the urban-adapted anthropophilic *Aedes aegypti* mosquito vector. Co-infection of arboviruses has been reported widely^14^, including between ZIKV and CHIKV, which is thought to enhance vector transmission^15^ and ZIKV pathogenicity^16^. The differential diagnoses of such arboviral infections are complex given their common early-stage presentation in patients. Molecular diagnostics exhibit very high specificity, however, these are only sensitive during the acute stages of infection and can be logistically prohibitive in low-resourced endemic settings^17^. With this, serology has formed an integral role in arbovirus diagnostic and surveillance strategies^18,19^, although, viral antigens from related species can result in immune response cross reactivity, which can confound assay specificity^20,21^. The use of non-structural proteins, such as *Flavivirus* NS1, in antibody detection assays for disease surveillance has been reported widely, and found to exhibit reduced cross reactivity^22,23^. Further, methods for increasing immunoassay specificity exist. These include blockade-of-binding and double antigen binding (DAB) assays, both of which detect all immunoglobulin (Ig) isotypes and have been shown to reduce non-specific antibody detection, specifically in DENV endemic regions^24–26^.

Cabo Verde has experienced numerous recent epidemics, including dengue fever (2009), Zika (2015) and malaria (2017)^27–29^. With respect to ZIKV, in November 2015, public health surveillance in Cabo Verde identified an increase in patients presenting with rashes, conjunctivitis, and myalgia^30^. By the time the outbreak concluded in May 2016, 7580 suspected cases of ZIKV infection and 18 cases of ZIKV congenital syndrome were recorded^31^, with 1.4% of the population (size 531k) infected. The estimated reproductive rate (R_0_) was 1.9 (95% CI 1.5–2.2)^31^. Praia, the capital city, had the greatest reported rate of ZIKV transmission in the country. Phylogenetic analysis has shown that Cabo Verdean 2015/2016 isolates cluster closely with those sourced from Brazil, suggesting that ZIKV was introduced from the Americas, and the outbreak was not of African origin^28^. Serological investigations on suspected ZIKV patients (n = 1226) recruited from clinics during the epidemic revealed that 226 (18%) were confirmed ‘recent infections’ by PCR or IgM positive assays, and 311 (25%) samples were IgG or plaque reduction neutralisation test (PRNT) positive^28^. An analysis of *Ae. aegypti* mosquitoes (n = 816) collected across Praia suggested there was low-level Zika virus circulation in mosquitoes (< 0.5%) shortly after the outbreak (August–October 2016)^32^.

Here, we present the findings from a serological surveillance study performed in Cabo Verde with convenience samples collected in and around Praia shortly after the conclusion of the ZIKV outbreak in 2016. By combining a panel of arbovirus antigens in an enzyme-linked immunosorbent assay (ELISA) and equivalent commercial solutions, with the analysis of extensive metadata representing a cross-section of the population in Praia, we infer rates of arbovirus seroconversion following the ZIKV outbreak on Cabo Verde, and identify risk-factors for ZIKV seropositivity.

## Materials and methods

### Study site and sample collection

This study was carried out in the city of Praia located on the island of Santiago, the region with the highest prevalence of vector-borne diseases in Cabo Verde. The municipality of Praia has an area of 102 km^2^, has a population of 142,009 and is divided into 88 localities^33^. To compare transmission across high and low prevalence regions, two locations, Plateau (1019 inhabitants) and Tira Chapéu (5785 inhabitants) were selected for this study. These locations reported the fewest and the most suspected Zika cases, according to the data from the Praia Health Delegacy. Residents from the islands of Fogo, Santiago, Boa Vista, Sal, and Brava were also included in this study but were all sampled at the collection centres in Praia.

Blood (5 ml) samples were collected for 7 weeks (August 24, 2016, to October 12, 2016), after the final ZIKV case was reported in Plateau and Tira Chapéu. These samples were obtained at the Health Center in Tira Chapéu and at the Health Department in Plateau. Blood samples were collected by venipuncture and transported same day to the UniPiaget laboratory for separation into serum and plasma. Before initiating sample collection, an awareness campaign was carried out in the two localities, with door-to-door home visits and distribution of information material. All individuals who participated in this study gave informed consent for sample and data collection. The data collection form contained three sections: personal characteristics (e.g., age, occupation), infectious disease history, and Zika symptoms. Ethical approval for this study was given by the Cabo Verde National Ethics Committee in research for health (ref. CNEPS 28/2016). All methods were performed in accordance with the regulations associated with the ethics approval. The study design is summarised in Fig. S1.

### Arbovirus indirect ELISA

Participant sera samples were tested for IgG antibodies against ZIKV (Native Antigen Company (NAC): ZIKVSU-NS1), DENV 1–4 (NAC: DENVX4-NS1-100), YFV (NAC: YFV-NS1-100) NS1 and CHIKV E1 (NAC: CHIKV-E1-100) protein antigens. In addition to the standard indirect NS1 ELISA, we employed a commercial DAB assay to increase assay specificity, given the expected high dengue seroprevalence. Samples were defrosted from − 80 °C storage at 7 °C overnight. Storage conditions for each plate were monitored and plates were defrosted at the same time and did not exceed 3 defrost cycles. Each 96 deep-well serum plate was centrifuged at 7 °C at full speed for 10 min. Lipaemic samples were identified, and the lipid layer was aspirated. Following a checkerboard titration for each antigen and a subset of samples, a serum dilution of 1:400 was identified as the optimal analyte concentration and an antigen coating concentration of 1 μg/ml for NS1 proteins and 2 μg/ml for CHIKV E protein was determined to provide optimal assay sensitivity. The antigens were coated on to HBX 96 well ELISA plates (Thermo: 3355) at the aforementioned concentrations in freshly prepared carbonate-bicarbonate buffer pH 10.6 overnight at 7 °C. Plates were blocked in 150 μl phosphate buffered saline + 0.05% tween (PBST) and 1% nonfat-dried milk (NFDM) at room temperature for 3 h and washed 5 times in PBST. Serum was diluted in 1% NFDM and 50 μl was pipetted on to each plate with ZIKV, DENV, YFV and CHIKV positive controls as well as negative controls for the four respective antigens and incubated at room-temperature for three hours and washed. 50 μl of secondary goat anti-Human IgG (H + L) secondary HRP antibody (Thermo: #A18805) was applied at a 1:7000 dilution (PBST) to each well and the plates incubated at room temperature for two hours and washed. Finally, 100 μl of tetramethylbenzidine substrate (TMB) (tebu-bio: TMBW-1000-01) was applied to each well and incubated at room temperature in low-light conditions for 15 min, stopped with 50 μl 1 M H_2_SO_4_ then plates were read at 450/620 nm.

### ZIKV double antigen binding (DAB) assay

HBX 96 well ELISA plates (Thermo: 3355) were coated with 1 μg/ml of ZIKV NS1 (NAC: ZIKVSU-NS1) covered overnight at room temperature and were washed for 5 cycles (PBST 200 μl) with an automated 96 well plate washer. Blocking was performed with 200 μl 3% BSA in DPBS for one hour at room temperature. Samples were diluted at a 1:100 ratio in to 0.5% BSA in DPBS with 0.2% tween and 100 μl applied to each well using a 12-channel automated multichannel pipette, followed by a 30-min room-temperature incubation on an orbital plate shaker at 800 rpm and washed, as above. 100 μl of biotinylated ZIKV NS1 (NAC) diluted in 0.5% BSA in DPBS with 0.2% tween was added to the plate and incubated for 30 min on an orbital plate shaker at 800 rpm. 100 μl of polystreptavidin HRP conjugate (NAC) in 0.5% BSA in DPBS with 0.2% tween was added and incubated for 30 min on an orbital plate shaker at 800 RPM and washed. Finally, 100 μl of TMB substrate (tebu-bio: TMBW-1000-01) was applied to each well and incubated at room temperature in low-light conditions on an orbital plate shaker at 800 RPM for 15 min, stopped with 100 μl 1 M H_2_SO_4_ then plates were read at 450/620 nm.

### Assay comparison

To further validate the IgG ZIKV and DENV ELISA assays, we tested a random subset of participants (n = 84), using an additional commercial indirect NS1 ELISA for ZIKV (ZG) and DENV (DG) IgG and IgM (EI 2668-9601). Samples were prepared as above, and the kit used as per manufacturer’s instructions. Each assay was adjusted using either our own or manufacturer's internal negative controls. The analysis of optical density (OD) correlation was performed and plotted with GGally^34^, with Spearman’s rank correlation coefficient (r_s_).

### Statistical models

We applied a Gaussian mixture model to classify samples into serological positive, intermediate, and negative groups. This involved the application of an expectation–maximisation algorithm to estimate the model parameters and to identify the most suitable number of Gaussian components using the Scikit-learn GMM package^35,36^. The optimum number of components were chosen using Akaike’s (AIC) and Bayesian (BIC) Information Criteria. Cut-offs between groups were determined by the 0.95 posterior probabilities for each component per model and used to classify titre ODs for further analysis (Fig. S2). Intermediate and negative groups were combined and compared to the positive group for the analysis of risk factors. Logistic regression models were applied to assess the association between ZIKV seropositivity and risk factors (e.g., age, gender, location, symptoms, and other serologically determined or self-reported flavivirus infections), summarised by odds ratios and their 95% confidence intervals.

## Results

### Patient cohort demographics and ZIKV risk

**Figure 1:**
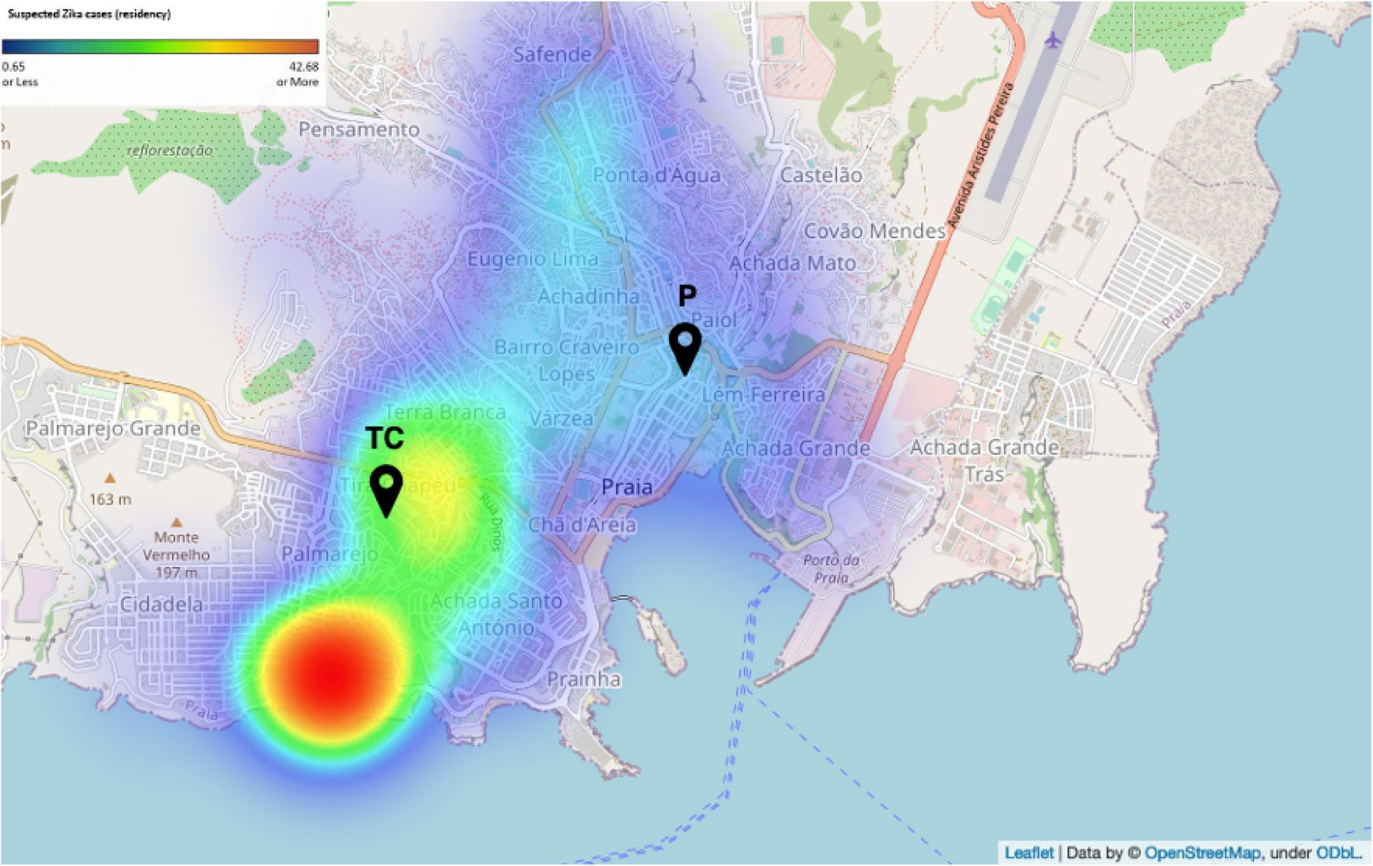
Geospatial distribution of suspected Zika infections in Praia, Cabo Verde. Data extracted and geocoded from the government’s surveillance programme. Cases recorded between October 2015 to April 2016. Collection centres in Praia indicated by TC (Tira Chapéu) and P (Praia). The map was generated by OpenStreetMap under ODbL and Folium (https://python-visualization.github.io/folium/).

A total of 732 sera samples were collected in Tira Chapéu (n = 395) and Plateau (n = 337) study centres between August 24, 2016, and November 4, 2016, of which a random subsample (n = 431, 58.9%) were processed (Fig. S1, Table 1). The median age of the 431 participants was 35 years (range 20–72 years), and the majority were female (n = 272, 63.1%). Of the 207 women aged between 20 and 44 years, 74 (35.7%) were pregnant. The participants with self-reported ZIKV infections (suspected infections) (6.7%) and symptoms of Zika virus disease (ZVD; rash, myalgia, nausea, or fever; 6.7%), had minimal overlap (n = 1). For self-reported infections, ZIKV was in lower prevalence than that of historical DENV infection (21.3%) and greater than malaria (1.9%). The median tympanic temperature of participants was 36.1 °C (range 23.3–37.7 °C), most of which were within the established normal range for this method (35.4–37.8 °C)^37^. Most participants reside in Praia (Santiago Island, n = 341, 79.1%) and on São Vicente Island (n = 31, 7.2%), which have high population densities and a high prevalence of ZIKV cases (n = 2480, October 2015 to April 2016; 79% Praia) (Table S1). Geospatial analysis of ZIKV surveillance data (n = 2480 cases) indicated that the majority of the suspected Zika infected population live in or around Tira Chapéu, one of the study sites in Praia (Fig. 1). The other Praia study site, Plateau, had a comparatively lower, but still relatively high ZIKV incidence during the epidemic (Fig. 1).

**Table 1:** Study characteristics.

Characteristic	N (median)	% (range)
Age (years)	35	20–72
Female	272	63.1
**Location**		
Praia	341	78.9
Sao Vicente	31	7.2
Other	59	13.9
Self-reported Zika	29	6.7
Self-reported malaria	8	1.9
Self-reported dengue	92	21.3
Body temperature (°C)	36.1	32.3–37.7
Any Zika symptoms*	29	6.7
Zika serology**		
Negative	193	44.7
Intermediate	191	44.3
Positive	47	10.9
Dengue serology**		
Negative	224	52.0
Intermediate	153	35.5
Positive	54	12.5
Yellow fever serology**		
Negative	255	59.2
Intermediate	175	40.6
Positive	1	0.2
Chikungunya serology**		
Negative	363	84.2
Intermediate	57	13.2
Positive	11	2.6

*A rash, myalgia, nausea, or fever; **based on a 3 components mixture model.

### Assay comparison

On a random subset of samples from our cohort (n = 84/431; 19.5%) we compared 3 commercial assays (ZIKV IgG indirect ELISA (ZG); DENV indirect IgG ELISA (DG); and ZIKV total Ig DAB ELISA) alongside an unmodified in-house ZIKV NS1 IgG indirect IgG ELISA and DENV NS1 indirect IgG ELISA using commercially acquired recombinant antigens. The strongest correlations were between the ZIKV and DENV NS1 IgG ELISA (Spearman’s r_s_ = 0.80) and the DG and ZG assays (r_s_ = 0.80), whereas the correlation between ZIKV DAB and DG assays was lower (r_s_ = 0.54). The ZIKV commercial assays were highly correlated with the ZIKV NS1 assay (DAB: r_s_ = 0.72; ZG: r_s_ = 0.71). We observed a reduction in DENV NS1 IgG ELISA positive samples classified as ZIKV positive when the ZIKV DAB assay (n = 3, 33% of DENV positive) was compared to the ZG assay (n = 8, 88% of DENV positive) (Figs. S3, S4). With this, we chose the ZIKV DAB as our primary ZIKV assay for the classification of the larger dataset (n = 431).

In addition to the IgG assays, we screened the subset for ZIKV and DENV IgM using additional ZG and DG IgM assay variants. The ZIKV DAB assay, which detects all isotypes, can infer the presence of IgM (or IgA) by an observable differential with the ZIKV NS1/ZG IgG assay. We found no samples that exhibited elevated anti-ZIKV IgM or IgM/IgA using any of the respective assays.

### ZIKV and other arbovirus serology results

Analysis of the 431 participant sera samples revealed that ZIKV, DENV 1–4, YFV and CHIKV seropositivity was 10.9%, 12.5%, 0.2% and 2.6%, respectively (Table 1). Almost a quarter (23.1%; 19/82) of ZIKV or DENV seropositive participants were found to be reactive to both DENV-NS1 (1–4) and the ZIKV-DAB assay. Of those, 63.1% (12/19) self-reported as having had either ZIKV or DENV infections, 5.2% (1/19) reported both, and 31.6% (6/19) no infections. The majority of ZIKV seropositive samples (63.8%) were collected in Tira Chapéu, consistent with the high ZIKV transmission reported in the geospatial analysis (Fig. 1). The distribution of DENV seropositive participants was spread across study centres (Tira Chapéu 46.2%, Plateau 38.8%, outside Praia 12.9%). Low levels of CHIKV seropositivity were observed (2.6%) with only a single participant YFV IgG positive. The latter findings are in line with the Ministério da Saúde e da Segurança Social epidemiological report, in which there were no reported YFV cases from 2015 to 2019^38^. The median age of all ZIKV, DENV and CHIKV seropositive participants (37 years; range 23–65 years) was higher than those seronegative (34 years, range 20–72 years) (Wilcoxon test P = 0.009). There was an association between self-reported ZIKV infection and seropositivity (Chi-square test, P < 0.001), as well as between DENV self-reporting and its seropositivity (Chi-square P = 1 × 10^–7^), but no evidence of association between self-reported ZIKV and DENV (P = 0.746).

There were high correlations between ELISA assay ODs (Fig. S5), especially between the two ZIKV assays (in-house ZIKV NS1 and ZIKV DAB, r_s_ = 0.74), in-house ZIKV NS1 and DENV NS1 (1–4) (r_s_ = 0.70) and DENV and YFV (r_s_ = 0.51). The correlation between ZIKV DAB and DENV NS1 (r_s_ = 0.34) was lower than for the NS1 assay, reflecting the greater specificity of ZIKV DAB methodology. Other high correlations (abs(r_s_) > 0.4) were between CHIKV and YFV infections, and there were low correlations between ELISA assay ODs and age (max. |r_s_| < 0.2) (Fig. S5).

### Risk factors and correlates of ZIKV seropositivity

Univariate analysis revealed several risk factors potentially associated with ZIKV seropositivity (positive 47 vs. non-positive 384), including DENV seropositivity (odds ratio (OR) 6.766, P < 0.001) and self-reported ZIKV (OR 6.213, P < 0.001) (Table 2). There was marginal evidence of ZIKV seropositivity associations with (higher) age (OR 1.025, 95% CI 0.998–1.051, P = 0.07), self-reported DENV infection (OR 1.303, 95% CI 0.998–1.051, P = 0.046) and CHKV seropositivity (OR 3.205, 95% CI 0.998–1.051, P = 0.094). There were no associations with sex, location or ZVD associated symptoms (P > 0.287) (Table 2). These risk factor results were robust to their inclusion in a multivariate model, except self-reported CHKV (P = 0.574) and DENV infection (P = 0.349), where the latter is correlated with DENV seropositivity.

**Table 2:** Odds ratios (ORs) for risk factors for Zika positivity (n = 47) versus non-positivity (n = 384).

Risk factor	Zika non +ve N (median)	Zika non +ve % (range)	Zika +ve N (median)	Zika +ve % (range)	OR	95% CI	P-value	AOR	95% CI	P-value
Age (years)	34	20–72	39	23–63	1.025	0.998–1.051	0.068	1.029	1.000–1.060	0.048
Male vs. female	239	62.2	33	70.2	0.699	0.352–1.325	0.287	0.654	0.314–1.361	0.256
Praia vs. other	301	80.2	39	83.0	1.199	0.564–2.860	0.658	–	–	–
Any Zika symptoms*	27	7.0	2	4.3	0.588	0.093–2.053	0.478	–	–	–
Dengue +ve	35	9.1	19	40.4	6.766	3.409–13.326	3.3 × 10^−8^	6.783	3.128–15.101	1.6 × 10^−6^
Yellow fever +ve	1	0.3	0	0	–	–	–	–	–	–
Chikungunya +ve	8	2.1	3	6.4	3.205	0.998–1.051	0.094	–	–	–
Self-reported Zika	18	4.7	11	23.4	6.213	2.660–14.043	1.4 × 10^−5^	4.889	1.913–12.493	9.1 × 10^−4^
Self-reported dengue	80	20.8	12	25.5	1.303	0.998–1.051	0.046	0.671	0.292–1.546	0.349

*AOR* adjusted odds ratios estimated by a multivariate model that includes all listed risk factors, *CI* confidence intervals; *a rash, myalgia, nausea, or fever.

## Discussion

Cabo Verde has been the location of several infectious disease outbreaks in the past 12 years, involving DENV, ZIKV and malaria infections. Within this historically high transmission setting, our study focused on a 3-month period after the recent Zika outbreak in 2016 and applied ELISA assays to perform a serological based assessment of the prevalence of ZIKV and other arbovirus diseases. We selected the DAB methodology as our primary ZIKV assay as it exhibited a reduction in the classification of DENV NS1 positive samples as ZIKV positive, when compared to the other indirect ELISA methodologies with known ZIKV-DENV cross reactivity.

Analysis of 431 participant sera samples revealed ZIKV and DENV (1–4) seropositivity in excess of 10%. Our study consisted of only adults, and predominantly women, with a high incidence of pregnancy (37.3%). We hypothesise that women are also more likely to visit medical clinics than men, with differences exacerbated due to concerns arising from the causal link between ZIKV infection and congenital Zika syndrome (similar to^39^). Our logistic regression modelling approach adjusted for gender and identified a significant link between greater risk of ZIKV seropositivity and increasing age. For the duration of the study, poster advertisements were placed encouraging the public to engage with the study. While we believe we have captured a balanced demographic in our cohort, a bias toward participants who suspect they may have been exposed to ZIKV may exist.

Our cohort consisted of primarily residents in the municipality of Praia (Santiago Island, 79.1%), the urban capital region with the greatest reported transmission of the archipelago, and São Vicente (7.2%) another island 266 km away from Praia. Additionally, participants reported as residing on Fogo, Boa Vista, Sal, and Brava islands (6.3%). The ZIKV vector, *Ae. Aegypti* thrives in urbanised environments, and therefore, is well positioned for arbovirus transmission in such areas^40,41^. This supposition is reflected in our study, in that the municipalities of Praia and São Vicente had the greatest number of ZIKV and DENV cases and were the two most densely populated municipalities in Cabo Verde.

In our cohort, 6.7% of participants self-reported with a ZIKV infection, of which 37.9% were ZIKV seropositive, which is consistent with another study in Cabo Verde that estimated 43.8% of self-reported suspected cases were ZIKV positive from PCR, IgM or IgG assay assessments^28^. Similarly, 29.3% of our participants who self-reported dengue infection were DENV seropositive, consistent with a report in Brazil (n = 20,880, 26.0–32.5%)^42^. Interestingly, there was a weak overlap between reporting of ZIKV symptoms and self-reported suspected ZIKV infection. It may be that participants recall of multiple symptoms is weaker than that of a single diagnosis. Further, the ambiguous presentation of ZVD in the context of a pandemic may act to confound epidemiological studies of this nature and should be interpreted only with robust supporting data.

CHIKV transmission has been reported to both enhance transmission and pathogenicity of ZIKV infections^15,16^. CHIKV is not included in the ‘Priority conditions and diseases under epidemiological surveillance’ programme^38^. We have observed low seropositivity of CHIKV in this cohort compared to that reported in Brazil^16^. While there have been no confirmed reports of autochthonous cases of CHIKV in Cabo Verde, this result emphasises the possibility of an introduction event occurring, especially given the number of seropositive (exposed) participants. This possibility is further compounded by the demonstrated competence of the local *Ae. Aegypti* vector population for the transmission of arbovirus pathogens.

Despite the more recent ZIKV outbreak, DENV seroprevalence was marginally greater. The DENV outbreak occurred in 2009 with 25,088 suspected cases, more than three times that of the ZIKV burden^43^. Since then, there have been low levels (36 cases, 2015–2019) of autochthonous DENV2 and DENV4 transmission^38,44^. In addition, there have been reports of secondary ZIKV infections activating memory anti-DENV B and T-cell effector responses through antigenic ‘original sin’ mechanisms^45,46^. These mechanisms potentially explain the high anti-DENV optical densities, and the significant concordance between the non-specific NS1 indirect ZIKV and DENV assays. Despite the optimisation of the commercial DAB and EI ZIKV NS1 assays, the possibility of ZIKV/DENV false-positives due to cross-reactive antibody responses is a study limitation. The application of PRNT assays would mitigate these confounding effects, however, they are not always possible and applicable in epidemiological contexts.

Overall, our findings provide new insights into the dynamics of a ZIKV outbreak in Africa, introduced from Brazil. Cabo Verde’s proximity to mainland Africa and close links with Brazil make it an involuntary trans-Atlantic hub for the introduction of infectious diseases to new continents. Our survey has provided a snapshot of arbovirus seropositivity across Cabo Verde, detailing demographics, and testing assays, while emphasising the requirement for sustained epidemiological surveillance to reduce the burden of future outbreaks, and potential pandemics.

## Supplementary Information

Supplementary Information.
