# Supplementary material for: Sero-epidemiological study of arbovirus infection following the 2015–2016 Zika virus outbreak in Cabo Verde
Source: Sci Rep. 2022 Jul 9;12:11719. doi: 10.1038/s41598-022-16115-4 (PMC9271056; doi:10.1038/s41598-022-16115-4)
Supplement: Supplementary file 1 — Supplementary Information. [file 41598_2022_16115_MOESM1_ESM.docx]

**Appendix**

**Table S1. Geographical data for our study cohort and from the Government of Cabo Verde’s surveillance programme (Govt. survey).** ZIKV and DENV positive (+ve) are represented as a percentage of total positive participants

| Civil municipality | Cohort (*n*) | Cohort (%) | ZIKV  +ve  (%) | dengue +ve  (%) | Govt.  Survey  (N) | Govt. survey  (%) | Population density*** (pop/km^2^) |
| --- | --- | --- | --- | --- | --- | --- | --- |
| Praia* | 341 | 79.1 | 83.0 | 83.6 | 1964 | 79.2 | 1384.1 |
| São Vicente | 31 | 7.2 | 10.6 | 3.6 | 83 | 3.3 | 326.5 |
| Santa Cruz* | 13 | 3.0 | 0.0 | 3.6 | 20 | 0.8 | 222.9 |
| Ribeira Grande de Santiago* | 11 | 2.6 | 4.3 | 3.6 | 143 | 5.8 | 55.6 |
| São Domingos* | 8 | 1.9 | 2.1 | 0.0 | 0 | 0.0 | 31.0 |
| Other** | 27 | 6.3 | 0.0 | 5.5 | 270 | 10.9 | - |

* Santiago Island; **Fogo, Santiago, Boa Vista, Sal, Brava islands; *** from 2021 census ^33^.

**Figure S1**

**Study design.** Overview of sample collection and analytical pipeline.

**
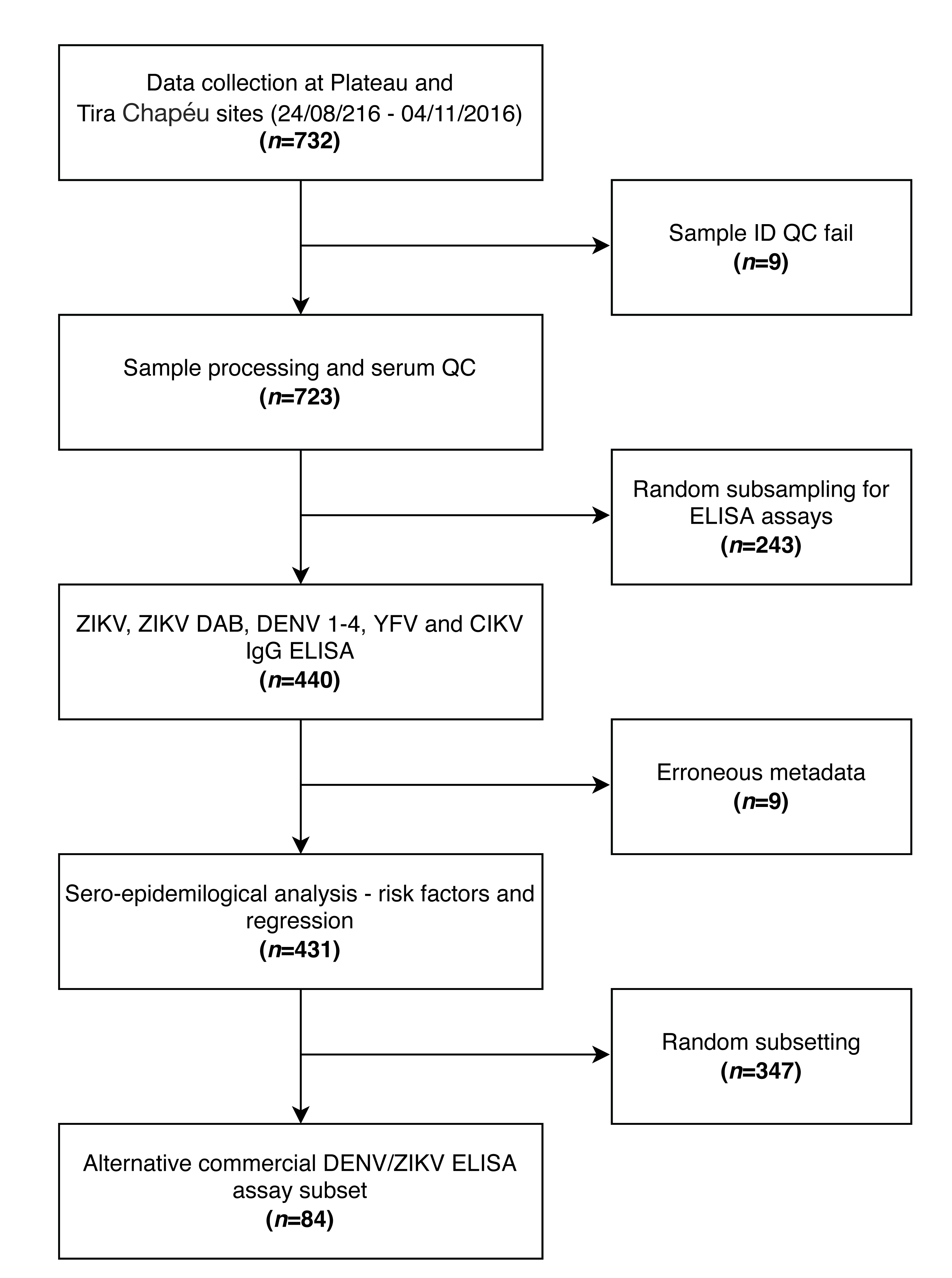
**

**Figure S2. Gaussian mixture model classification of ELISA results**.

**
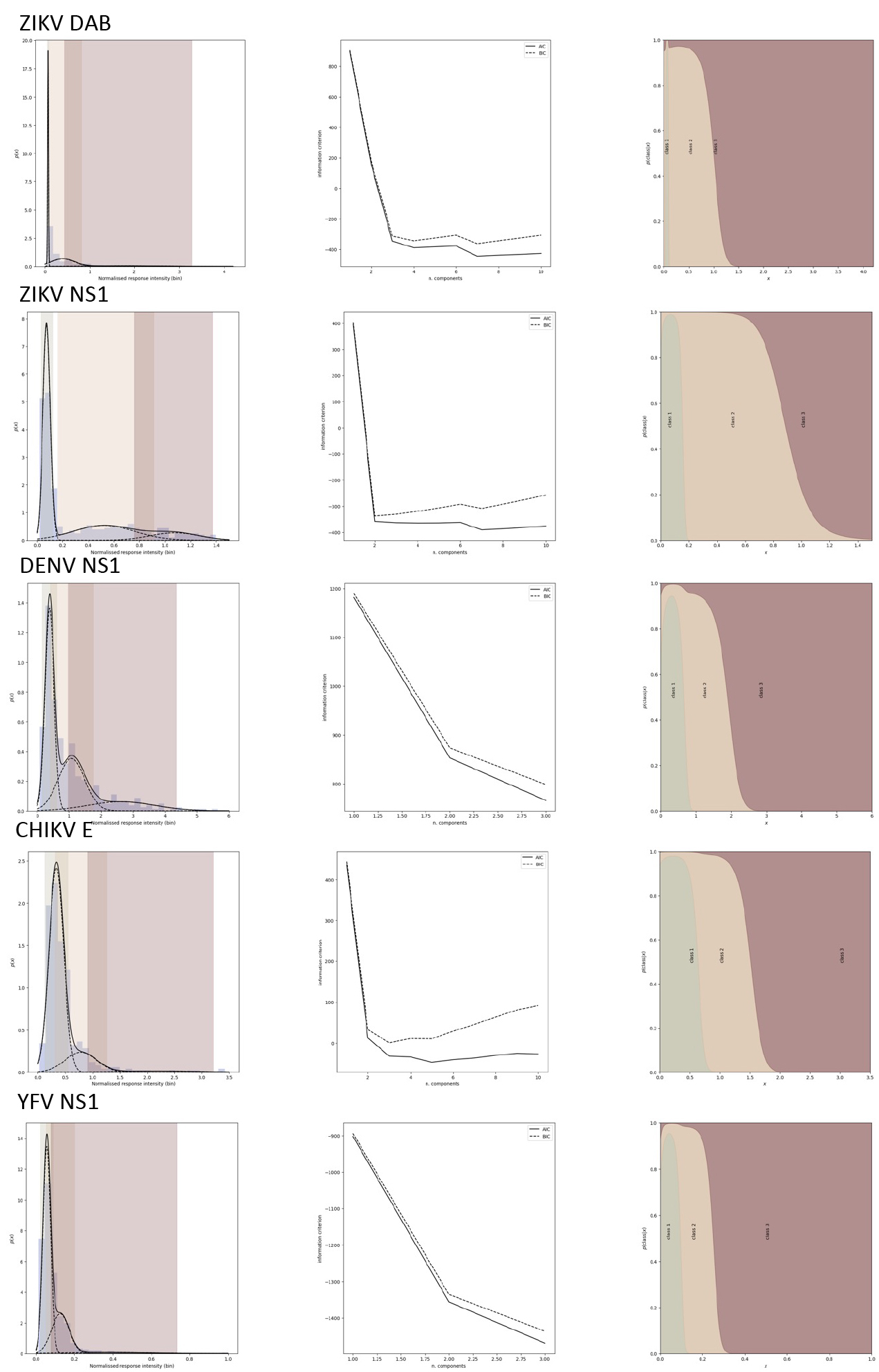
**(Left) Frequency distribution with Gaussian components plotted. (Centre) Akaike's (AIC) and Bayesian (BIC) Information Criteria to determine optimal component count. (Right) Curves defining posterior values for probability of classification in each component.

**Figure S3. Comparison of arbovirus serological assays on 84 samples.**

(Upper) Spearman’s correlation coefficient (r_s_). (Diagonal) Frequency distribution histogram of processed assay optical densities. (Lower) Scatterplot of processed assay optical densities with a fitted LOESS curve. Colours of points: (Red) ZIKV + DENV NS1 assay negative; (Green) ZIKV NS1 assay positive; (Blue) DENV NS1 positive; (Purple) ZIKV + DENV NS1 positive. Colours of dashed lines: (Red) Negative cut-off; (Black) Positive cut-off; (Green) ZIKV NS1 positive; (Blue) DENV NS1 positive.

**
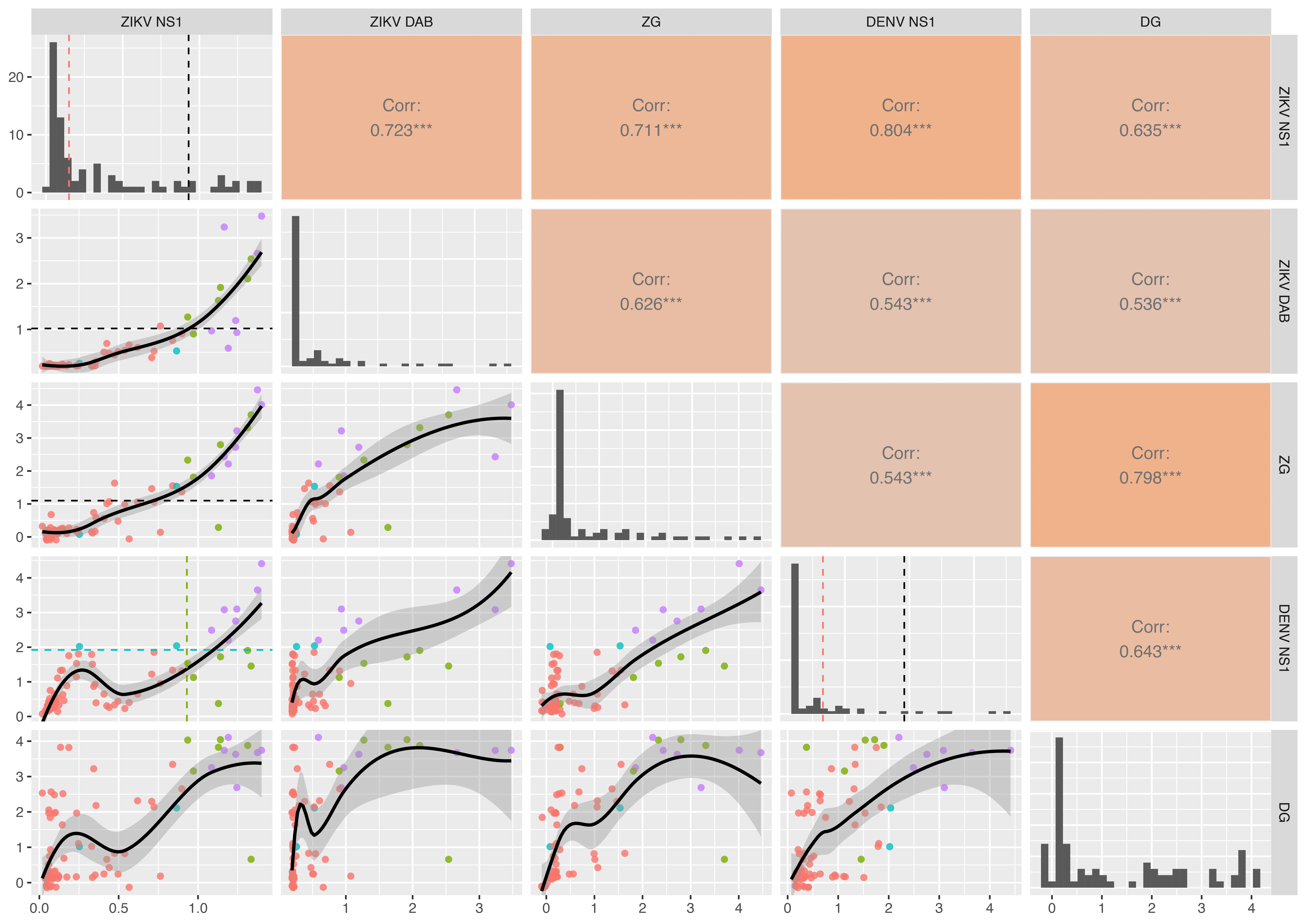
**

**Figure S4. Intersection in seropositivity between alternative ZIKV (ZG) and DENV (DG) commercial and respective NS1 ELISA assays (n=84).**

**
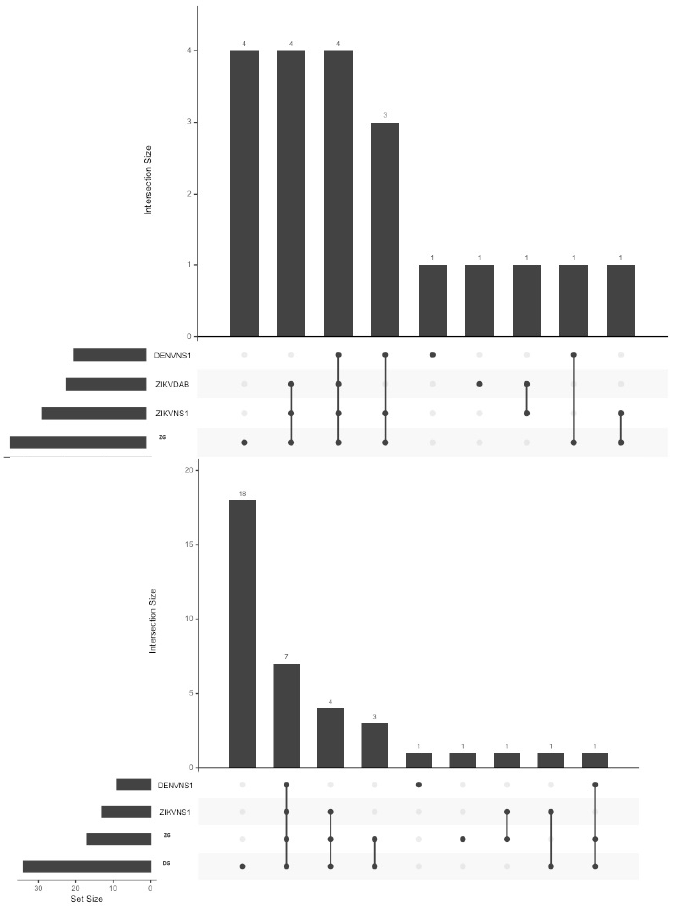
**

**
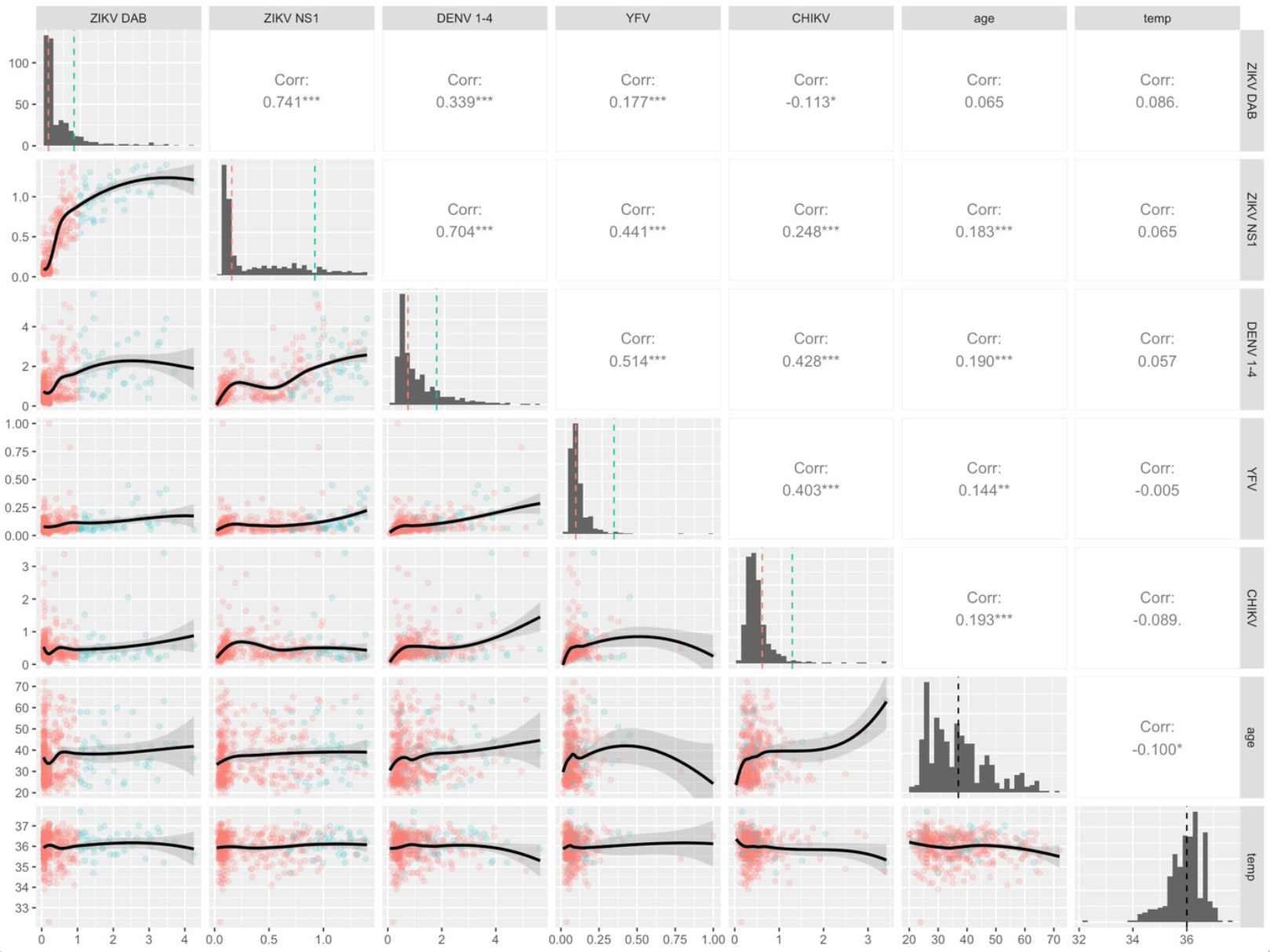
Figure S5. Correlations between processed optical densities of ELISA assays, age, and body temperature**

YFV Yellow fever, ZIKV Zika virus, DENV dengue virus, Temp = body temperature, Age in years. Correlation was tested with Spearman’s rank (Corr = r_s_). Red and green dashed lines denote ELISA cut-offs for -ve/int/+ve. Black dashed line indicates the dataset average; *** P-value < 0.05
